# Rapid In Situ Coating of Covered Stents with Highly Tough, Biocompatible Membrane for Emergency Coronary Artery Perforation

**DOI:** 10.3390/biom15111608

**Published:** 2025-11-17

**Authors:** Yuan Ji, Mingyue Fan, Bing Li, Guolin Gao, Zaixing Jiang

**Affiliations:** State Key Laboratory of Advanced Inorganic Fibers and Composites, School of Chemistry and Chemical Engineering, Harbin Institute of Technology, Harbin 150001, China; 19B925124@stu.hit.edu.cn (Y.J.); 23B325001@stu.hit.edu.cn (M.F.); libzz@hit.edu.cn (B.L.)

**Keywords:** covered stents, highly tough membrane, biocompatibility, coronary artery perforation, in situ coating

## Abstract

Covered stents have made a significant contribution to managing coronary artery perforation (CAP). Biocompatibility and toughness are critical properties for the covering membrane of covered stents. The mismatch between covered stents and patient coronary arteries in the clinic restricts the application of covered stents for emergency CAP. The ability to rapidly in situ coating of the stent at the rescue scene has so far been elusive, especially for small-diameter coronary artery covered stents. Here, we investigate a rapid coating technology of covered stents with polyvinylidene fluoride (PVDF)/dibutyl phthalate (DBP) covering membrane for CAP. The highly tough membrane and the short coating timeframe make it possible to prepare the covered stent suitable for patients with emergency CAP. In vitro cell assays demonstrated the excellent biocompatibility of the covering membrane. Moreover, in vivo evaluation in a rabbit model demonstrated successful delivery of the covered stent through the sheath system and effective sealing of vascular perforation.

## 1. Introduction

Coronary artery perforation constitutes a severe complication of percutaneous coronary intervention (PCI), associated with significantly increased mortality rates [1,2,3]. Coronary artery perforation may precipitate rapid clinical deterioration into hemopericardium and cardiac tamponade, culminating in fatal outcomes due to acutely impaired cardiac filling [4,5,6]. Consequently, time-sensitive emergency intervention serves as a critical determinant for patient survival [7,8,9]. Endovascular implantation of covered stents constitutes a primary therapeutic intervention for coronary artery perforation [10,11,12]. In the management of a coronary artery perforation, a covered stent is delivered to the site of vascular injury via a balloon-tipped catheter. The balloon inflation achieves stent expansion, apposing the covered stent firmly against the vessel wall to seal the perforation [13]. The covering membrane of the stent thereby provides a physical barrier, preventing life-threatening extravasation of blood into the pericardial space [14]. The toughness and biocompatibility of the membrane material are critical for the effective implantation of the covered stent [15].

Advances in the coating strategies of covered stents have facilitated the application of the polymer membrane onto vascular stents. However, the coating technology has been impeded by significant hurdles, including the technical intricacies of the manufacturing process and suboptimal mechanical properties of the covering membranes [16,17,18]. The suboptimal toughness and large outer diameter of covered stents pose significant challenges for their delivery into the coronary artery [15]. One of the limitations of covered stents is the serious shortage of covered stents with appropriate clinical coronary artery [19]. Currently, polytetrafluoroethylene, polyurethane, and polylactic acid have been utilized as covering membranes for coronary stents [15,20,21]. The coating methods generally include electrospinning, weaving, and bonding [22,23]. The utility of these methods is limited by the redundant and complex procedures, and covered stents are prone to situations such as membrane rupture and detachment. While the shorter preparation timeframe and higher property requirements of the membrane make it extremely difficult to prepare covered stents at the rescue scene. Frequently, a covered stent suitable for patients is unavailable during the CAP repair procedure. It misses the timeframe for rescue and significantly increases patient mortality rates.

In response to this clinical challenge, we have developed an in situ coating technique to cover a stent at the rescue scene, which is aimed at bridging the gap between the advancements in covered stent manufacturing technology and the exigent requirements of clinical rescues. With its excellent biocompatibility, chemical stability, and excellent mechanical properties, PVDF holds significant potential for expanded applications through appropriate modification [24,25,26], which is widely used in the biomedical field [27,28,29]. Furthermore, PVDF exhibits excellent processability and moldability, and enables rapid membrane formation. The PVDF/DBP covering membrane was prepared using PVDF as the matrix. The excellent toughness of the covering membrane makes it suitable for the common types of vascular stents clinically. Furthermore, the results of the in vitro cell experiment and in vivo animal experiment confirmed that covered stents in this study have great potential for clinical application. The key benefits of this technique are the short coating timeframe and simple operation, which allow for the coating of covered stents suitable for CAP patients during surgery.

## 2. Materials and Methods

### 2.1. Materials

N, N-dimethylacetamide (DMAc, 99%) and PVDF (Mw = 400,000) were procured from Macklin (Shanghai, China). Phosphate-buffered saline (PBS) was obtained from Genom (Hangzhou, China). DBP (99%) and Ethanol were obtained from Aladdin (Shanghai, China). The MTT cell proliferation kit was acquired from Roche (Basel, Switzerland), and flow cytometry apoptosis kits were procured from BD Pharmingen (San Diego, CA, USA). Biotronik AG, Inc. (Berlin, Germany) supplied metal coronary stent systems. Glutaraldehyde was purchased from Senbeijia (Nanjing, China). All reagents were used as supplied, without further purification. Deionized water, purified using a water purification system, was employed in all experiments.

### 2.2. Preparation of Covered Stent

#### 2.2.1. Preparation of Casting Solution

For the preparation of the casting solution, the PVDF (10 wt%, 15 wt%, 20 wt%, 25 wt%, 30 wt%) was added to DMAc. The blend was agitated at 60 °C for 4 h until the PVDF had fully dissolved. To improve the toughness of the membrane, DBP was embedded in the PVDF molecular chain. PVDF pellets and DBP (0 wt%, 5 wt%, 10 wt%, 15 wt%, 20 wt%, 25 wt%) were added to the DMAc. The mixture was agitated at 60 °C for 4 h until completely dispersed. Subsequently, the casting solution was sealed and stored at room temperature for 24 h to defoam completely.

#### 2.2.2. Coating of the Coronary Stent

To ensure uniform coating adhesion, the bare metal stent was submerged in the casting solution under ultrasonication for 1 min. By the non-solvent-induced phase separation method, the stent coated with the casting solution was placed vertically into the coagulating bath (pure water) at 30 °C until the casting solution was completely solidified. Remove the water from the membrane surface.

### 2.3. Mechanical Property of the Membrane

All mechanical properties were measured using an electronic testing machine (SANS CMT 8102, Shenzhen, China). The casting solution (150 μm) was uniformly spread onto the glass sheet and subsequently submerged in the 30 °C deionized water for membrane formation. Membrane samples were subjected to tensile testing at a constant speed of 2.0 mm/min until rupture.

### 2.4. Chemical Characterization of the Membrane

The molecular structure of the membrane was analyzed via the Fourier Transform Infrared spectroscopy (Nicolet is50, Thermo Fisher Scientific, Waltham, MA, USA). Analysis was performed in the range of 600–4000 cm^−1^. The elemental composition of the samples was determined using X-ray photoelectron spectroscopy (AXISULTRA DLD, JP, Thermo Fisher Scientific, Waltham, MA, USA).

### 2.5. Differential Scanning Calorimetry (DSC)

The thermal properties of the samples were evaluated using a differential scanning calorimeter (NE-TZSCH STA 449F3, Selb, Germany). The sample was heated at 30–700 °C at 10 °C/min under a nitrogen flow of 100 mL/min.

### 2.6. Scanning Electron Microscopy (SEM)

All microstructure photos were taken using SEM based instruments (SUPRA55, ZEISS, Oberkochen, Germany). The covered stent was fabricated using the procedures described in Section 2.1 and Section 2.2. Following curing, the membrane was peeled axially. The membranes were air-dried and subjected to gold sputtering. Both outer and inner surfaces of the membrane were analyzed using SEM.

### 2.7. Storage Modulus

The rheometer was used to record the storage modulus curve of the membrane samples as frequency. The membrane sample was cut into discs (25 mm in diameter) and positioned on a parallel plate fixture attached to the rheometer (AR2000 ex, TA, New Castle, DE, USA). The storage modulus (G′) was determined by frequency from 0.1 to 100 Hz at 37 °C.

### 2.8. In Vitro Cell Experiment

This experiment utilized human umbilical vein endothelial cells (HUVECs, iCell, Shanghai, China) and human umbilical artery smooth muscle cells (HUASMCs, Procell, Wuhan, China). HUVECs and HUASMCs were cultured at 37 °C with 5% CO_2_, respectively. The membrane underwent sterilization at 121 °C under high pressure for 20 min.

#### 2.8.1. MTT Assay

The biocompatibility of the membrane was assessed employing the MTT assay. In this experiment, membranes were co-cultured with HUVECs and HUASMCs, respectively. A density of 5 × 10^4^ cells/mL of cells was suspended in a 96-well plate. Concurrently, the 100 μL cell suspension was added to each well. The sample was added after the cells were attached to the wall. Cells were incubated for 24 h at 37 °C. In the control group, cells were grown lacking the membrane. Then, each well was treated with 10 μL of MTT solution and cultured at 37 °C for 4 h. Following this, each well was treated with 100 μL of PBS and cultured for 10 min. Absorbance readings were taken at 570 nm, with the assay performed 6 times.

#### 2.8.2. Scratch Test

The cell migratory capacity was evaluated via the scratch assay. To prepare the cells for experimentation, they were trypsinized following growth to the logarithmic phase. After adjusting cell concentration, suspensions were plated in a 6-well plate according to 1 × 10^5^ cells/well in 5% CO_2_ at 37 °C for 24 h.

The pipette tip was used to make vertical scratches in the culture wells of each group and was washed with PBS 3 times and then replaced with the fresh medium. Samples were added to the experimental group. Following incubation at 37 °C in 5% CO_2_ for 0 h, 12 h, and 24 h, cells were imaged under a confocal laser microscope. For each time point, more than 5 representative micrographs were acquired and analyzed using ImageJ 1.8.0 software.

#### 2.8.3. Apoptosis Assay

Flow cytometry was employed to quantify apoptosis. A density of 1 × 10^6^ cells/mL of HUVEC cells was suspended. Concurrently, the 100 μL cell suspension was added to each well of the 6-well plate. Following fresh medium replacement, samples were transferred to wells and incubated for 24 h. HUVECs were then washed twice with PBS and trypsinized. After centrifugation, supernatants were removed. A 100 μL Binding Buffer was added to the wells and the cells were stained with 5 μL Annexin V-FITC. The mixture was then incubated for 30 min at room temperature, protected from light. Cells were then stained with 5 μL propidium iodide (PI) and incubated for 5 min at room temperature, protected from light. After adding 400 μL PBS, suspensions were filtered through a cell strainer and immediately analyzed by flow cytometry. Each group of samples was tested in triplicate.

### 2.9. Hemocompatibility

The membrane samples were cut into square pieces. A 150 μL rabbit-derived platelet-rich plasma (PRP) was added on the surface of the membranes and incubated at 37 °C for 1 h. The membranes were washed three times with PBS. The membranes were fixed in 2.5% glutaraldehyde for 1.5 h and then rinsed three times with PBS solution again. After dehydration in gradient ethanol 30% to 100%, the morphology of platelets adhered to the samples was observed by SEM.

### 2.10. In Vivo Covered Stent Implantation in Rabbit

A male New Zealand white rabbit, approximately 3 months old and weighing about 3.6 kg, was utilized for the study. Before surgical intervention, the rabbit was anesthetized with 3% pentobarbital sodium. Angiography was performed to determine the appropriate vessel segments for experimentation. A rigid guide wire was implanted through the rabbit carotid artery and inserted into the arterial wall of the rabbit abdominal aorta. The successful creation of vessel perforation was confirmed by observing blood leakage via angiography. The covered stent prepared in this paper was implanted from the rabbit carotid artery by catheter and transported to the site of the abdominal aortic vessel perforation. Animal studies were approved by the Experimental Animal Management and Welfare Ethics Committee of the First Affiliated Hospital, Harbin Medical University (IACUC No. 2022150).

### 2.11. Statistical Analysis

The results are represented as the mean ± standard deviation. Statistical analysis was conducted by the *t*-test. The significance was defined as 0.05.

## 3. Results and Discussion

### 3.1. Design of Covering Membranes

**Figure 1:**
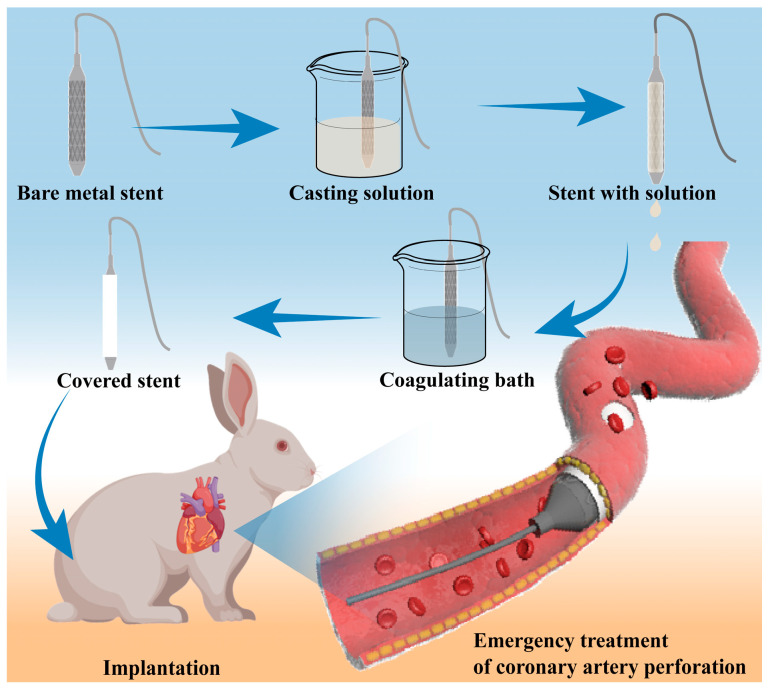
Schematic diagram of the covered stent coating process and the rescue of coronary artery perforation.

Rapidly applying a tough and biocompatible membrane to the surface of vascular stents remains a significant challenge. The short duration of the preparation and the suitability of covering membranes are critical. To our knowledge, the coating method of covered stents with a processing time under several minutes at the rescue scene has not been reported. In situ curing by non-solvent induced phase separation can achieve a rapid covering membrane on the bare vascular stent surface [30]. The simpler preparation process and the more rapid membrane curing make this way suitable for the covered stent preparation at the rescue scene [31]. In this study, we examined bare metal stents as a representative case. Figure 1 shows the covered stent coating process and rescue procedure for coronary artery perforation.

**Figure 2:**
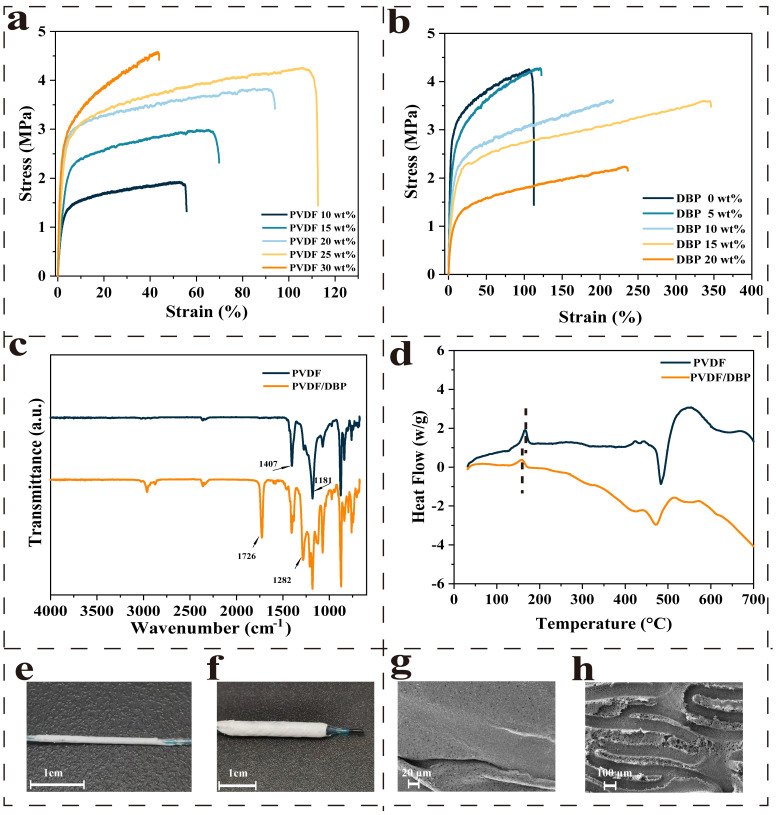
Material optimization, molecular structure, and morphology of the covered stent. (**a**) The mechanical properties of the PVDF membrane with different PVDF concentrations. Among them, the PVDF concentrations in the casting solution were10 wt%, 15 wt%, 20 wt%, 25 wt%, and 30 wt%, respectively. (**b**) Mechanical properties of the modified membrane. The additional amounts of DBP were 0 wt%, 5 wt%, 10 wt%, 15 wt%, and 20 wt%, respectively. (**c**,**d**) FTIR spectra and DSC curves of the membrane before and after modification. (**e**,**f**) Images of the covered stent before and after expansion in vitro. (**g**,**h**) SEM images of the outer and inner surfaces of the membrane of the covered stent before expansion.

Following coating with casting solution, the stent was subsequently immersed in a coagulating bath to form the membrane by NIPS. Among them, the rapid diffusion of solvent and non-solvent leads to phase separation of the membrane system, and its diffusion behavior can be explained by Fick’s second law. Biocompatibility and mechanical properties are the key determinants of the covering membrane. Figure 2a shows the tensile properties of the PVDF membrane, which is formed by the casting solution with different PVDF concentrations. The inset illustrates that the membrane elongation did not exceed 106% at the optimal PVDF concentration of 25%. While there is still a gap between this value and our expected minimum 150% elongation, which is the common expansion stent external diameter during percutaneous coronary intervention [32,33,34].

The small organic molecule was used to modify the PVDF matrix by external plasticization [35]. To investigate the correlation between membrane toughness and Dibutyl phthalate (DBP) concentration, the casting solutions were prepared with DBP additions of 5 wt%, 10 wt%, 15 wt%, and 20 wt%. The stress–strain curves of the modified membranes are presented in Figure 2b. The modified membranes exhibited significantly enhanced toughness across nearly all samples, with a strong correlation to DBP addition. The addition of DBP was determined to be 15% because the change in membrane toughness was the most apparent, where the membrane’s elongation reached 337.33% with an elevation of 218.51%. This may be because DBP molecules were inserted into the molecular chains of PVDF, reducing the intermolecular forces of the polymer, and thus, improving the free volume of the polymer chain. The greater free volume of polymer chains improved the deformability and tensile properties of the membrane system. When the PVDF with DBP was subjected to stress, DBP could disperse the stress and reduce the degree of stress concentration, thereby enhancing the fracture elongation of the material.

Figure 2c shows FTIR spectra before and after the modification of the membrane. The peaks near 1407 cm^−1^ and 1181 cm^−1^ are the characteristic peaks of the C-H and C-F bonds of PVDF, respectively. The peaks near 1726 cm^−1^ and 1282 cm^−1^ are the characteristic peaks of C=O and C-O of DBP, respectively. To investigate the effect of the DBP on the phase inversion temperature of the membrane system, DSC was conducted on the membranes. Figure 2d shows the DSC curves of the membranes before and after adding DBP. According to free volume theory, the decrease in membrane phase inversion temperature was shown after adding DBP because of the increase in the polymer system’s free volume [36]. DBP molecules hindered the formation of hydrogen bonds between PVDF chains, improving the freedom degrees of molecular chain movement.

With our easy way, one might expect to achieve a 100% success rate. Unfortunately, our in vitro experiments revealed that this method did not guarantee a 100% success rate. There was a persistent risk of membrane rupture upon stent expansion. During the phase inversion process, non-uniform coating of the casting solution on the substrate surface led to the formation of pores and structural defects. To address this, ultrasonic treatment was employed. This technique utilized the acoustic radiation force to eliminate entrapped air pockets at the solution–stent interface, thereby promoting superior wetting and uniform adhesion. The success rate of the coating of covered stents was greatly improved. Figure 2e,f show the photos before and after the in vitro expansion of the covered stent. Figure 2g,h show SEM images of the outer and inner surfaces of the membrane of the covered stent before expansion, offering insights into its morphology. The inner membrane architecture presented the stent’s pattern, confirming conformal adhesion.

**Figure 3:**
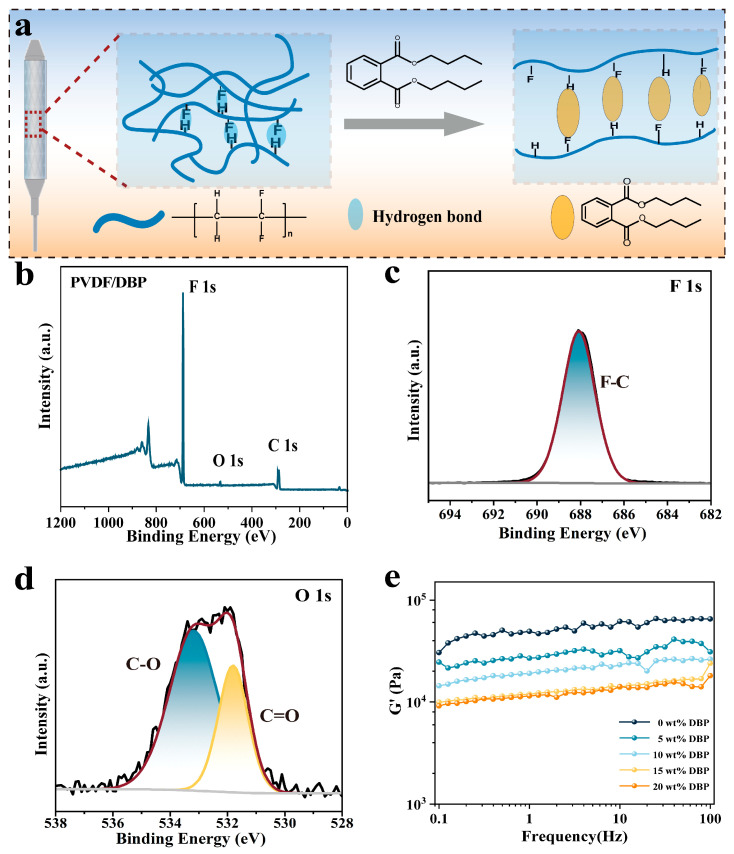
Covering membranes of covered stent characterization. (**a**) Membrane material modification mechanism diagram. (**b**) XPS full spectrum. (**c**,**d**) F 1s and O 1s XPS spectra of the modified membrane. (**e**) The storage modulus of the membranes with frequency.

To further analyze the molecular interactions in the membrane system, the elemental composition of the modified membrane was investigated. Figure 3a shows the mechanism of DBP modification of the PVDF system. Strong hydrogen bonding and dipole–dipole interactions exist between PVDF molecular chains. The insertion of DBP into the interchain spaces of PVDF occupies these polar sites and engages in dipole–dipole interactions with the PVDF molecules. This effectively increases the free volume between the chains, thereby facilitating the enhanced mobility of the PVDF chains. Figure 3b shows the XPS full spectrum of the PVDF/DBP membrane, including F 1s, O 1s, and C 1s peaks. Figure 3c shows the F 1s spectrum, and one of the fitted peaks belongs to C-F. Figure 3d shows the O 1s spectrum. The addition of DBP results in an obvious O peak in the XPS spectrum. It was allocated to the two peaks, which were ascribed to C=O bonds and C-O bonds. It was formed by the ester group in the DBP molecular structure of the modified membrane.

To quantify the kinetic changes in the membranes, we measured the change in storage modulus of the covering membrane before and after modification. Figure 3e presents the comparison of the storage modulus (G’) of the membrane before and after modification. Almost all kinetic behaviors of the modified membrane showed obvious changes. DBP addition exhibited a strong correlation with changes in membrane intermolecular interactions, resulting in a distinct reduction in the storage modulus. We considered several reasons for the dynamic change in the membrane. The small molecule DBP could be embedded into the polymer chains. DBP had a polar interaction with C-F in PVDF, which increased the spacing between PVDF molecular chains and reduced the hydrogen bonding and entanglement between the molecular chains. The dynamic changes in the covering membrane were consistent with the macroscopic mechanical properties.

### 3.2. Effects of the Membrane Materials on In Vitro Biocompatibility

**Figure 4:**
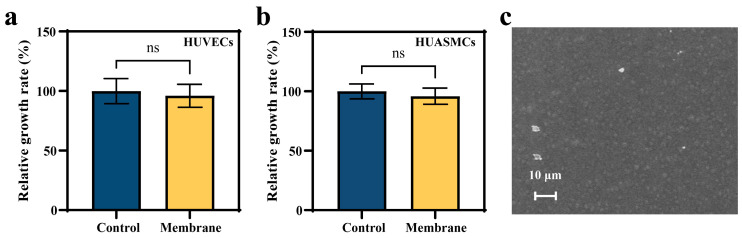
(**a**) The HUVECs’ relative growth rate by an MTT assay. Each group of samples was tested 6 times. (**b**) The HUASMCs’ relative growth rate by an MTT assay. (**c**) Morphologies of adherent platelets on the surface of the membrane. ns means not significant.

To correlate the membrane materials with biocompatibility, the proliferation, scratch, and apoptosis assays were conducted to provide comprehensive evaluations of cellular behavior [37]. We co-cultured samples with HUVECs and HUASMCs, respectively. Figure 4a,b show the proliferation results of HUVECs and HUASMCs after co-culture with the membrane for 24 h. The relative growth rates (RGR) of HUVECs and HUASMCs were (96.17% ± 9.67%) and (95.97% ± 6.76%), respectively. The MTT assay demonstrated that the membrane exerted no significant effect on cell activity. The result revealed that the RGR of the cells co-cultured with the membrane were above 90%, which was considered safe [38]. To verify the hemocompatibility of the membrane material, the platelet adhesion of the membrane was tested. Figure 4c shows the platelets adhered to the surface of the membrane sample. The results indicate that only a small number of platelets are attached to the sample surface, and they are not in an activated state [39].

**Figure 5:**
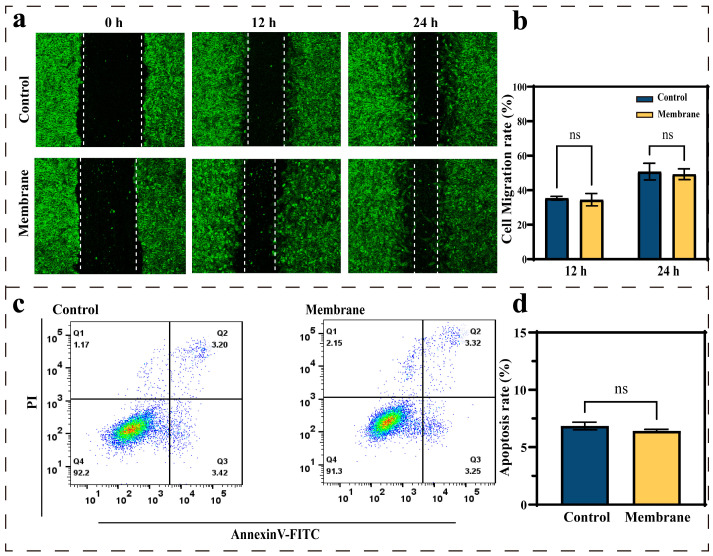
(**a**) HUVECs cell migration test. Images are collected every 12 h, each group of samples was tested in triplicate. (**b**) HUVECs migration rate. (**c**) HUVECs apoptosis results of the control group and the membrane group, and (**d**) the apoptosis rate of HUVECs. Each group of samples was tested in triplicate. ns means not significant.

To further investigate the biocompatibility of the covering membrane with the cells on the inner wall of blood vessels, the migration ability and apoptotic performance of HUVECs were characterized. Figure 5a presents microscopic images of cell migration in the control and membrane groups. Images were acquired every 12 h. The migration rates between the control group (50.75% ± 4.82%) and the membrane group (49.21% ± 3.17%) showed no significant difference after 24 h (Figure 5b). The results showed that membrane materials did not inhibit cell migration behavior. This indicated that the coating material did not impair the formation of endothelialization. Figure 5c shows the flow apoptotic result of HUVECs co-cultured with membranes. Figure 5d compares HUVECs apoptosis rates of the control group and the membrane group. There was a strong correlation between the membrane group (6.43% ± 0.13%) and the control group (6.86% ± 0.32%) at 24 h. The covering membrane did not induce excessive apoptosis of cells. These findings demonstrated that HUVECs populations co-cultured with membrane materials did not have significant physiological changes. Membrane materials exhibit a strong correlation with biocompatibility.

### 3.3. Covered Stent Deployment in the Rabbit Abdominal Aorta

**Figure 6:**
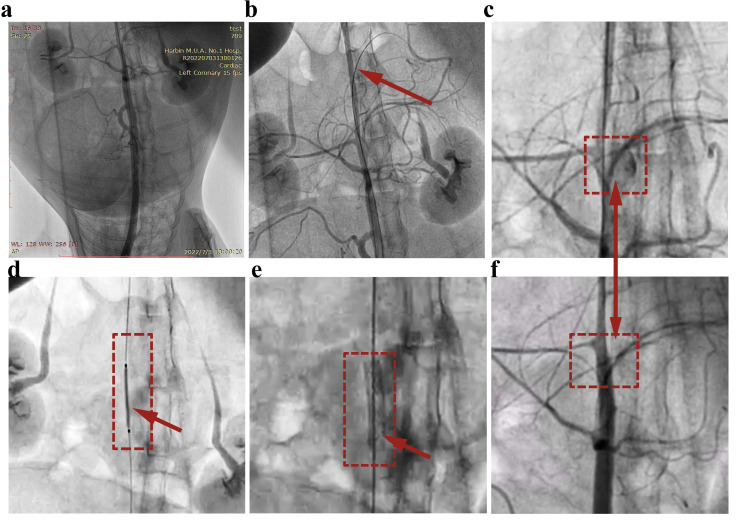
Digital angiography photos of in vivo animal experiments. (**a**) Abdominal aortography image of the animal. (**b**) Establishment of the CAP model. (**c**) Blood diffusion through the vessel. (**d**) Implantation of a covered stent. (**e**) Expansion of the covered stent. (**f**) The vessel is successfully sealed, and the blood outside the vessel is absorbed.

Buoyed by these promising findings, we advanced to test the efficacy of the covered stent developed in this research using animal subjects. Figure 6 shows angiographic photos at several time points during the animal experiment. Figure 6a,b show the establishment of the CAP model of the rabbit abdominal aorta. The most obvious effect of the CAP model is the diffusion of blood from the aorta to the outside of the vessel (Figure 6c). Figure 6d shows the successful implantation of the covered stent prepared in this study into the vascular perforation site. Expansion of the covered stent is visible after the balloon is pressurized (Figure 6e). Figure 6f shows that the blood stopped diffusing after the covered stent sealed the infected part, and the extravasated blood was gradually absorbed. The illustration shows the effective sealing of the perforation after the expansion of the covered stent. These observations in the in vivo animal experiments demonstrated the potential of the covered stent using our method for emergency CAP.

## 4. Conclusions

Covered stents constitute a critical therapeutic intervention for emergency coronary artery perforation, demonstrating vital utility in interventional cardiology. We investigated a rapid in situ coating technique of the covered stent with a highly tough and biocompatible membrane. The membrane elongation rate reached 337.33%, which is suitable for the common types of coronary artery stents in the clinic. It was confirmed that the material had good biocompatibility by in vitro cell experiments and platelet adhesion tests. By establishing an animal model of coronary artery perforation, it was verified that the stent covered using our method had clinical feasibility in the treatment of CAP. These findings demonstrated the significant potential of covered stent for emergency CAP. The advances of vascular stent coating reported here represent the enabling technologies for scientists to explore the preparation process of cardiovascular implants.

## Data Availability

The original contributions presented in this study are included in the article. Further inquiries can be directed to the corresponding authors.

## Abbreviations

The following abbreviations are used in this manuscript:

CAP	Coronary artery perforation
PCI	Percutaneous coronary intervention
PVDF	polyvinylidene fluoride
DBP	Dibutyl phthalate
NIPS	Non-solvent induced phase separation

## References

[1] Strycek M., Jaworski L., Polasek R., Tomasov P. (2023). Coronary artery perforation successfully treated with a second drug-eluting stent. J. Int. Med. Res..

[2] Shaikh R., Tomdio A.N., Lawson B.D. (2024). Management of Coronary Artery Perforation in a Patient with Stemi. JACC.

[3] Kandzari D.E., Sarao R.C., Waksman R. (2022). Clinical Experience of the PK Papyrus Covered Stent in Patients With Coronary Artery Perforations: Results From a Multi-Center Humanitarian Device Exemption Survey. Cardiovasc. Revascularization Med..

[4] Lemmert M.E., van Bommel R.J., Diletti R., Wilschut J.M., de Jaegere P.P., Zijlstra F., Daemen J., Van Mieghem N.M. (2017). Clinical Characteristics and Management of Coronary Artery Perforations: A Single-Center 11-Year Experience and Practical Overview. J. Am. Heart Assoc..

[5] Harnek J., James S., Lagerqvist B. (2020). Coronary Artery Perforation and Tamponade—Incidence, Risk Factors, Predictors and Outcomes From 12 Years’ Data of the SCAAR Registry. Circ. J..

[6] Danek B.A., Karatasakis A., Tajti P., Sandoval Y., Karmpaliotis D., Alaswad K., Jaffer F., Yeh R.W., Kandzari D.E., Lembo N.J. (2017). Incidence, Treatment, and Outcomes of Coronary Perforation During Chronic Total Occlusion Percutaneous Coronary Intervention. Am. J. Cardiol..

[7] Solomonica A., Kerner A., Feld Y., Yalonetsky S. (2020). Novel Technique for the Treatment of Coronary Artery Perforation. Can. J. Cardiol..

[8] Khan A., Kumar R., Ali R., Fatima K., Abid M., Ali R., Meheshwari G., Amin R., Hasan M., Kasi A.F.U.D. (2023). The prevalence, angiographic profile and clinical features, management, and outcomes of coronary artery perforation secondary to percutaneous coronary interventions in Pakistan: A retrospective cohort study. Ann. Med. Surg..

[9] Kinnaird T., Anderson R., Ossei-Gerning N., Cockburn J., Sirker A., Ludman P., de Belder M., Johnson T.W., Copt S., Zaman A. (2017). Coronary Perforation Complicating Percutaneous Coronary Intervention in Patients With a History of Coronary Artery Bypass Surgery: An Analysis of 309 Perforation Cases From the British Cardiovascular Intervention Society Database. Circ. Cardiovasc. Interv..

[10] Moroni F., Brilakis E.S., Azzalini L. (2021). Chronic total occlusion percutaneous coronary intervention: Managing perforation complications. Expert Rev. Cardiovasc. Ther..

[11] Harnek J., James S.K., Lagerqvist B. (2019). Very long-term outcome of coronary covered stents: A report from the SCAAR registry. Eurointervention.

[12] Barbero U., Cerrato E., Secco G.G., Tedeschi D., Belliggiano D., Pavani M., Moncalvo C., Tomassini F., De Benedictis M., Doronzo B. (2020). PK Papyrus coronary stent system: The ultrathin struts polyurethane-covered stent. Futur. Cardiol..

[13] Farhatnia Y., Tan A., Motiwala A., Cousins B.G., Seifalian A.M. (2013). Evolution of covered stents in the contemporary era: Clinical application, materials and manufacturing strategies using nanotechnology. Biotechnol. Adv..

[14] Giannini F., Candilio L., Mitomo S., Ruparelia N., Chieffo A., Baldetti L., Ponticelli F., Latib A., Colombo A. (2018). A Practical Approach to the Management of Complications During Percutaneous Coronary Intervention. JACC Cardiovasc. Interv..

[15] Secondo M.T.S., Rodrigues L.D., Ramos L.P.M., Bovolato A.L.C., Rodriguez-Sanchez D.N., Sobreira M.L., Moraes M.P.D., Bertanha M. (2022). Evaluation of Biointegration and Inflammatory Response to Blood Vessels Produced by Tissue Engineering-Experimental Model in Rabbits. Biomolecules.

[16] Morgan G.J., Ciuffreda M., Spadoni I., DeGiovanni J. (2018). Optimus covered stent: Advanced covered stent technology for complex congenital heart disease. Congenit. Heart Dis..

[17] Cohen S., Magal S., Yakov I., Sirabella E., Bitman A., Groisman G., Lotan C. (2018). Tissue processing techniques for fabrication of covered stents for small-diameter vascular intervention. Acta Biomater..

[18] Lee W.C., Hsueh S.K., Fang C.Y., Wu C.J., Hang C.L., Fang H.Y. (2016). Clinical Outcomes Following Covered Stent for the Treatment of Coronary Artery Perforation. J. Interv. Cardiol..

[19] Postalian A., Krajcer Z. (2021). Pushing covered stents to the limit. Catheter. Cardiovasc. Interv..

[20] Hou R.X., Wu L.G., Wang J., Yang Z.L., Tu Q.F., Zhang X.C., Huang N. (2019). Surface-Degradable Drug-Eluting Stent with Anticoagulation, Antiproliferation, and Endothelialization Functions. Biomolecules.

[21] Shen Y.H., Yu X., Cui J., Yu F., Liu M.Y., Chen Y.J., Wu J.L., Sun B.B., Mo X.M. (2022). Development of Biodegradable Polymeric Stents for the Treatment of Cardiovascular Diseases. Biomolecules.

[22] Chashmi F.S., Khakbiz M., Zahedi P., Kabiri M. (2022). Poly(lactic acid) Nanofibrous Scaffolds Containing Aspirin-loaded Zeolitic Imidazolate Frameworks: Morphology, Drug Release, Hemocompatibility and Shape Memory Studies. Fibers Polym..

[23] Yu Y.X., Song G.Z., Dai M.N., Li P.X., Xu J.M., Yin Y., Wang J.N. (2024). Tailoring silk-based covering material with matched mechanical properties for vascular tissue engineering. Sci. Rep..

[24] Chiu Y.S., Rinawati M., Chang L.Y., Aulia S., Shi P.C., Haw S.C., Huang W.H., Septiani N.L.W., Yuliarto B., Yeh M.H. (2025). High-dielectric TiO_2_-mediated g-C_3_N_4_ enhanced self-polarized PVDF hybridized film for highly sensitive wearable triboelectric pressure sensors. Chem. Eng. J..

[25] Bhaskar N., Basu B. (2023). Osteogenesis, hemocompatibility, and foreign body response of polyvinylidene difluoride-based composite reinforced with carbonaceous filler and higher volume of piezoelectric ceramic phase. Biomaterials.

[26] Azimi S., Golabchi A., Nekookar A., Rabbani S., Amiri M.H., Asadi K., Abolhasani M.M. (2021). Self-powered cardiac pacemaker by piezoelectric polymer nanogenerator implant. Nano Energy.

[27] Huang Z.X., Li L.W., Huang Y.Z., Rao W.X., Jiang H.W., Wang J., Zhang H.H., He H.Z., Qu J.P. (2024). Self-poled piezoelectric polymer composites via melt-state energy implantation. Nat. Commun..

[28] Hu R.H., Liu P., Zhu W., Guo X., Liu Z., Jin Z.X., Yang R.H., Han J.W., Tian H.L., Ma Y.M. (2025). Design, Fabrication, and Wearable Medical Application of a High-Resolution Flexible Capacitive Temperature Sensor Based on the Thermotropic Phase Transition Composites of PEO/PVDF-HFP/H_3_PO_4_. Adv. Funct. Mater..

[29] Abdulghani S., Mitchell G.R. (2019). Biomaterials for In Situ Tissue Regeneration: A Review. Biomolecules.

[30] Pullano S.A., Critello C.D., Bianco M.G., Menniti M., Fiorillo A.S. (2021). PVDF Ultrasonic Sensors for In-Air Applications: A Review. IEEE Trans. Ultrason. Ferroelectr. Freq. Control.

[31] Pochivalov K.V., Basko A.V., Ilyasova A.N., Lebedeva T.N., Yurov M.Y., Bronnikov S.V. (2023). Experimental phase diagram for the PVDF-DMAc-water ternary system with new topology: Method of construction, thermodynamics, and structure formation of membranes. Polymer.

[32] González N., Fernández-Berridi M.J. (2006). Application of Fourier transform infrared spectroscopy in the study of interactions between PVC and plasticizers: PVC/plasticizer compatibility versus chemical structure of plasticizer. J. Appl. Polym. Sci..

[33] Mannion A.M., Bates F.S., Macosko C.W. (2016). Synthesis and Rheology of Branched Multiblock Polymers Based on Polylactide. Macromolecules.

[34] Schneider J., Bourque K., Narayan R. (2016). Moisture curable toughened poly(lactide) utilizing vinyltrimethoxysilane based crosslinks. Express Polym. Lett..

[35] Zhou Q.H., Wang Z., Shen H.J., Zhu Z.Y., Liu L.P., Yang L.B., Cheng L.N. (2016). Morphology and performance of PVDF TIPS microfiltration hollow fiber membranes prepared from PVDF/DBP/DOP systems for industrial application. J. Chem. Technol. Biotechnol..

[36] Nechitailo V.S. (1992). About the Polymer Free-Volume Theory. Int. J. Polym. Mater. Polym. Biomater..

[37] Belibel R., Sali S., Marinval N., Garcia-Sanchez A., Barbaud C., Hlawaty H. (2020). PDMMLA derivatives as a promising cardiovascular metallic stent coating: Physicochemical and biological evaluation. Mater. Sci. Eng. C.

[38] Ai F.R., Liu T.W., Liu Y., Yang K., Liu Y.S., Wang W.Y., Yuan F.S., Dong L.N., Xin H.B., Wang X.L. (2018). A 3D printed wound cooling system incorporated with injectable, adsorbable, swellable and broad spectrum antibacterial scaffolds for rapid hematischesis processing. J. Mater. Chem. B.

[39] Tang H.Y., Li S.S., Zhao Y., Liu C.L., Gu X.N., Fan Y.B. (2022). A surface-eroding poly(1,3-trimethylene carbonate) coating for magnesium based cardiovascular stents with stable drug release and improved corrosion resistance. Bioact. Mater..

